# GPs´ decision-making - perceiving the patient as a person or a disease

**DOI:** 10.1186/1471-2296-13-38

**Published:** 2012-05-16

**Authors:** Malin André, Annika Andén, Lars Borgquist, Carl Edvard Rudebeck

**Affiliations:** 1 Centre for Clinical Research, Falun, Sweden; 2 Department of Medicine and Health Sciences, Family Medicine, Linköping University, Linköping, Sweden; 3 Department of Public Health and Caring Sciences – Family Medicine and Preventive Medicine, Uppsala University, Uppsala, Sweden; 4 Bergnäsets Vårdcentral, Luleå, Sweden; 5 Institute of Community Medicine, University of Tromsø, Tromsø, Norway; 6 Research Unit of Kalmar County Council, Kalmar, Sweden

## Abstract

**Background:**

The aim of this study was to analyse the clinical decision making strategies of GPs with regard to the whole range of problems encountered in everyday work.

**Methods:**

A prospective questionnaire study was carried through, where 16 General practitioners in Sweden registered consecutively 378 problems in 366 patients.

**Results:**

68.3% of the problems were registered as somatic, 5.8% as psychosocial and 25.9% as both somatic and psychosocial. When the problem was characterised as somatic the main emphasis was most often on the symptoms only, and when the problem was psychosocial main emphasis was given to the person. Immediate, inductive, decision-making contrary to gradual, analytical, was used for about half of the problems. Immediate decision-making was less often used when problems were registered as both somatic and psychosocial and focus was on both the symptoms and the person. When immediate decision-making was used the GPs were significantly more often certain of their identification of the problem and significantly more satisfied with their consultation. Rules of thumb in consultations registered as somatic with emphasis on symptoms only did not include any reference to the individual patient. In consultations registered as psychosocial with emphasis on the person, rules of thumb often included reference to the patient as a known person.

**Conclusions:**

The decision-making (immediate or gradual) registered by the GPs seemed to have been adjusted on the symptom or on the patient as a person. Our results indicate that the GPs seem to recognise immediately both problems and persons, hence the quintessence of the expert skill of the GP as developed through experience.

## Background

General practice is first line health care, in which the general practitioner (GP) manages patients with unsorted problems. Many problems are self-limiting and are never given definite diagnoses. One classic study indicated that many encounters were about self-limiting disorders, preventive services or psychosocial problems [1]. Thus outcomes as patient reassurance and better patient coping are important outcomes in general practice [2,3]. However, studies of GP’s consultations showed that only half of the health-affecting psychosocial problems were disclosed, which may result in problematic outcomes [4,5]. To secure the patient’s agenda in the consultation, the patient-centred method was developed for use in general practice, defined as the physician sharing the decisions with the patient and/or focusing on the patient as a person rather than solely on the disease [6,7]. In addition to learning the patient-centred approach, GPs have to be updated regarding clinical guidelines as well as being aware of early signs of a potentially serious disease. General practice thus operates at the point of intersection between health care as a medical-technological and a humanistic enterprise [8].

Managing the diversity of problems encountered puts high demands on GPs to use a variety of clinical decision-making strategies. In a study from general practice less than 50% of the cases resulted in certainty of a “known” diagnosis without further testing [9]. Although there is always some uncertainty at hand in any medical work, decisions still have to be made both regarding the nature of the problem and actions to be taken [10]. The limited time allotted for each consultation requires, if possible, rapid actions. In clinical reasoning two major ways of making decisions have been described [11-13]. One is immediate, inductive recognition, which is heuristic and largely experience-driven and sometimes called System 1 [14]. In contrast, the other model, sometimes called System 2, is slower, deductive, gradual and analytical [14]. The immediate, intuitive response to a specific situation characterises the expert. The perception of the situation is a crucial element in the holistic, rapid process of any expert determination [15]. Intuitive judgement has been subject for discussion, as it sometimes is marvellous and sometimes flaws [12]. Heneghan reports of four triggers for a diagnosis that often occurs early in the consultation, which get refined during the later refinement stage: spot diagnosis, self-labelling, the presenting complaints and pattern recognition [9]. GPs in Sweden described heuristics or rules of thumb as useful and necessary tools in their everyday work. Rules of thumb with two different purposes have been identified: to simplify categorisation of the problem to a disease and to make the consultation patient-centred [16,17].

Only few studies have explored the decision-making strategies of GPs concerning the unselected problems encountered [18,19]. The aim of this study was to analyse GPs’ use of decision making strategies (immediate or gradual), in relation to problem characteristics (somatic and/or psychosocial problem) and the process of consultation (weight given to symptoms and/or person).

## Method

GPs working all over Sweden and also interested in research and development in general practice were contacted through an informal network and asked to participate. Sixteen GPs were recruited for the study, 8 men and 8 women. These GPs worked in primary health-care centres in both rural areas and small towns. Most of the GPs were between 50 and 60 years of age with more than 20 years of working experience in general practice. They were asked to fill out questionnaires on 25 consecutive consultations. Informed consent for participation in the study was obtained from the GPs. The questionnaire was presented as an Excel file mailed to the GPs.

The questionnaire concerned the characteristics of the patient and the problems presented and the process of the decision-making of the GP (Table 1). All questions consisted of closed answers except the diagnosis and rule of thumb, which were given in free wording. A short written set of instructions was enclosed. The main patient problem was to be registered. However, when several problems were brought up during the consultation it was optional to register more than one. The definition of a rule of thumb was “a mental pattern used during the consultation, action-oriented and which comes to mind automatically”.

**Table 1:** Questionnaire with criteria describing the consultation

**Criteria**	**Variable**
*The patient*	Age
	Sex
*Problem characteristics*	Diagnostic code and diagnosis in words
	Previously known problem/new problem according to the medical records
	Somatic (yes/no)
	Psychological or psychosocial (yes/no)
*Main emphasis of the consultation*	The symptom (yes/no)
	The patient as a person (yes/no)
*Patient´s knowledge*	The patient had identified the problem him/herself (yes/no)
*Decision-making*	Immediate/gradual
	Certain/uncertain
	Rule of thumb used (yes/no)
	Rule of thumb in words
	I (the GP) was satisfied (yes/no/don’t know)

The questionnaire was piloted and changed several times for clarity and simplicity in order to be applicable in the GP’s surgery. The completed questionnaires were checked for inconsistencies by MA. The results of the study was discussed in subsequent seminars with GPs, both those involved in the study and not.

No ethical approval was needed as the participants were GPs and not patients.

### Statistics

The Chi-square test was used to test for associations. P < 0.05 was regarded as significant. Odds ratio (OR) was calculated in a logistic regression analysis with 95% confidence intervals (CI). The data was analysed with SPSS 15.0.

## Results

The GPs registered 15–30 patients each. Thus 366 patients with 378 problems in total, 94.2% somatic problems and 31.7% psychosocial problems were registered. Hence, there was some overlap, and 25.9% of the problems were registered as both somatic and psychosocial (Table 2). Most frequently only one problem was registered for each consultation, but in 12 consultations two different problems were registered. The majority of patients were between 15 and 64 years of age, 6.1% were children younger than 15 years and 30.4% were over 64 years of age.

**Table 2:** Number and percentage of registered items in relation to problem characteristics

	**Somatic problem only**	**Somatic and psychosocial problem**	**Psychosocial problem only**	**Total**
n (%)	258 (68.3)	98 (25.9)	22 (5.8)	378 (100)
Female patient	136 (52.7)	58 (59.2)	15 (68.2)	209 (55.3)
Known problem	84 (32.5)	64 (65.3)	15 (68.2)	163 (43.1)
*Process of consultation*				
Main emphasis on symptom	244 (94.6)	72(73.4)	8 (36.4)	324 (85.7)
Main emphasis on person	61 (23.6)	78 (79.6)	19 (86.4)	158 (41.8)
Patient had identified the problem him/herself	145 (56.2)	52 (53.1)	18 (81.8)	215 (56.9)
*Decision-making of the GP*				
Immediate	153 (59.3)	36 (36.7)	12 (54.5)	201 (54.7)
Certain	200 (77.5)	70 (71.4)	17 (77.3)	287 (75.9)
Rule of thumb used	93 (36.0)	24 (24.5)	7 (31.8)	124 (32.8)

**Figure 1:**
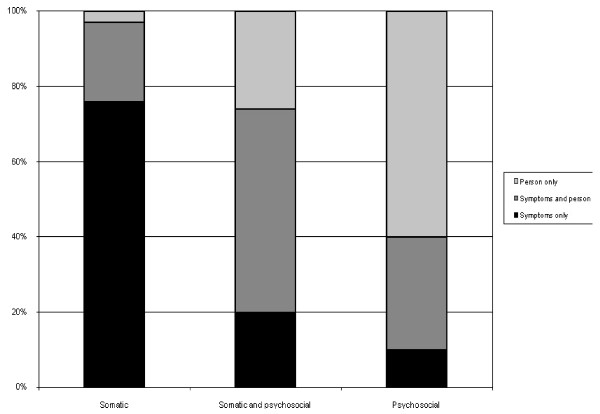
**Consultations labelled as somatic, somatic and psychosocial, and psychosocial only.** Emphasis given to symptoms only, symptoms and person and person only.

Immediate decisions were registered in 54.7% of the problems (Table 2). When immediate decision-making was used the GPs more often felt certain about their identification of the problem (p < 0.001) and the use of a rule of thumb was registered more often (p = 0.03). When the GPs noted that the patient had identified the problem, immediate decision-making was registered more often (p < 0.001), and the GPs felt more certain of their identification of the problem (p < 0.001). In exclusively somatic problems, emphasis was most often given to the symptom (94.6%) whereas in exclusively psychosocial problems most often emphasis tended to be placed on the patient as a person (86.4%), (Figure 1). In eight problems emphasis was neither on the symptom nor on the person and most of them concerned administrative issues.

The consultations were defined according to the identified problem and how the GP experienced the emphasis of the consultation. Thus identified categories, when consisting of more than 10 problems, were used in further analyses. In this way six categories with a focus gradually changing from somatic to more and more personal and psychosocial issues were formed (Table 3). Immediate decision-making dominated in somatic problems when emphasis was given to symptoms and in psychosocial problems when the focus was on the patient as a person. Immediate decision-making was registered significantly less frequently when the problems were registered as somatic and psychosocial and emphasis was given to both symptom and person (OR 0.26 CI 0.13-0.53) (Table 3). In somatic problems, where the focus was on symptoms, only 25.1% of the patients had consulted previously for the same problem. In comparison, the patient’s problem was significantly more often known when the emphasis was given to the patient as a person (p < 0.001).

**Table 3:** Characteristics of the problem and main emphasis in the consultation; immediate versus gradual decision-making

	**Odds ratio**	**Confidence interval (95%)**
Somatic problem only, emphasis given to symptoms	1.00, ref	
Somatic problem only, emphasis given to symptoms and person	0.59	0.31-1.09
Somatic and psychosocial problem, emphasis given to symptom	0.36	0.13-1.02
Somatic and psychosocial problem, emphasis given to symptom and person	0.26	0.13-0.53
Somatic and psychosocial problem, emphasis given to person	0.99	0.39-2.54
Psychosocial problem only, emphasis given to person	1.77	0.45-6.86

Rules of thumb in consultations registered as somatic with emphasis on symptoms only, did not include any reference to the individual patient, as exemplified by two diagnoses and registered rules:

"“Knee injury, cruciate; Hemarthrosis of knee + injury, serious.”"

"“Joint pain; No joint swelling, neither tenderness nor redness – innocuous joint pain”."

In consultations registered as psychosocial with emphasis on the person, rules of thumb often included reference to the patient as a known person, for example:

"“Hypochondria; This patient always has somatoform problems, but remain attentive.”"

"“Myalgia; This patient almost always develops problems when she is assigned dull, repetitive work tasks.”"

In contrast in consultations registered as both somatic and psychosocial with emphasis both on symptoms and person the rules of thumb often included reference both to the individual patient and the somatic problem at hand.

"“Diabetes; Problematic individual, make a follow-up appointment.”"

"“Urethritis; Patient extremely thorough and fastidious, orderly and anxious, probably has no STD”."

## Discussion

The vast majority of problems were registered as somatic, but in 25.9% the problem was at the same time registered as psychosocial. Only a small fraction (5.8%) was registered as psychosocial problems only. Immediate decision-making dominated in consultations with somatic problems and emphasis given to symptoms and in psychosocial problems when the focus was on the patient as a person. The rules of thumb registered for somatic problem where emphasis was given to the person included no reference to the patient as a person. In contrast, the rules registered in psychosocial problems when the focus was on the patient as a person contained reference to the patient as a person. When immediate decision-making was used the GPs were significantly more often certain of their identification of the problem as well as more satisfied with the consultation.

Different methods have been used to study the decision-making of physicians [20]. As the aim of this study was to analyse the clinical decision-making strategies in an every-day clinical setting of GPs as perceived by the GPs only data registered by the GPs was used. Had the study included for examples transcripts of the consultation, it would have been possible to validate the reported emphasis given to symptom or person in the consultation. On the other hand face validity is high as the GPs made the registrations in direct connection to the consultation. Moreover the results were recognised when discussed by GPs in subsequent seminars.

The registration of problem as somatic or psychosocial applied to the assessment of the problem, not to the management part of the consultation. The GPs were not randomly selected, but through informal personal contacts. However, they had not been involved in earlier research concerning decision-making and were well spread out over the country representing different parts of primary care all over Sweden. In Sweden only 10% of the GPs are the age of 30 to 40 due to limited recruiting during two decades, which explains the narrow age span of GPs (The Swedish Medical Association, personal communication). Moreover, Swedish GPs have longer consultations and their patients have fewer appointments per year than GPs in other Western countries [21]. This fact may restrict comparisons with other countries.

The internal loss of data was small (less than 10 registrations). Filling out a questionnaire after each consultation may have prevented hindsight bias, which is a prominent limitation of “think–aloud” protocol and stimulated recalls [22]. Although the questionnaire was piloted several times no qualitative validation was carried out to find out how the wordings were perceived. However, the consistency of data indicates fairly similar understanding among the GPs. No widely-accepted definition of “rule of thumb” exists and the type of statements registered varied. Our typology of the consultations into categories was based on intuitive order Thus these preliminary results must be confirmed by further more elaborated studies.

Each GP was requested to register 25 consecutive consultations, and the mixture of registered diagnoses indicates that the consultations were not selected. In a GP’s practice the diagnoses express different kinds of insights depending on the character of the diagnoses and depending on the different ways they are reached. Diagnoses are thus the products of situations involving individuals, rather than of a uniform, scientific logic [23].

We do not know whether the association between identified problem and process of consultation was attributable to the consulting style of the GP or whether the patient’s agenda elicited an adequate response from the GP. Winefield et al. stated that differences between individual GPs may influence how their patients express their needs at the consultation [18]. Previous studies have shown that most GPs tend to keep to their own working style independent of the problem presented [24]. The main issue is to adapt the decision-making to the task at hand, which is apparent with regard to the wide range of problems encountered in general practice.

In almost all consultations (94%) a somatic problem was registered. However, in one third of the consultations psychosocial aspects of the problems were also identified. In a study from Scotland the classification of consultations was done on the basis of the patient’s reason for making the appointment [19]. Thus 39.6% of the consultations were classified as biomedical problems, 21.3% as social problems, 9.6% as psychological problems and 20.1% as complex problems. Winefield et al. classified the appointments according to transcribed consultations (43% of the consultations straightforward, 33% psychosocial, and 24% complex) [18]. Our findings are in line with these studies, which supports the validity of our results given the methodological limitations.

In more than half of the consultations decision-making was registered as immediate. Immediate decision-making is a result of experience and builds on recognition. Rules of thumb earlier described from GPs were possible to generalise to specific symptoms or diseases [16]. In this study GPs also acknowledged automatically retrieved knowledge pertinent to a specific patient. Ethnographic studies have described how socially-constructed mindlines, which include also context specific knowledge, are used in this rapid almost unconscious decision-making in general practice [25]. In addition, emotions seem to have a guiding effect in the non-analytical decision-making [13]. In situations where the clues are not immediately given or perceived by the GP, the choice of strategy is based on the GP’s empathetic grasp of roughly what the problem may be about [26]. The GPs used immediate decision-making to a higher extent both when the problem was somatic, where most patients had not consulted earlier for their problem and when the problem was psychosocial and already known. An explanation, supported by the wording in the registered rules of thumb, might be that in relation to somatic problems GPs have recognised symptoms, whereas in relation to the psychosocial problems the individual patient as a person was recognised.

When immediate decision-making was used the GPs felt more certain of their identification of the problem and were more often satisfied with the consultation. This might be expressions of overconfidence, which is described as one of the risks in intuitive decision-making [12]. Although uncertainty is inherent in all medical practice, it may be most pronounced in general practice. However, in our study the GPs registered certainty in their identification of the current problem in three quarters of the consultations. This high figure may be partly explained by the fact that physicians seem reluctant to disclose their uncertainty when talking to patients [27], and that GPs most often responded to ambiguous symptoms by ignoring them [28]. Since office consultations are characterised by time pressure, decisions have to be made quickly, both to categorise the problem and to take action. Previous studies indicate that the decision on management sometimes precedes the decision of diagnosis in general practice [29]. Limiting the search for alternatives makes sense from an action perspective, while careful consideration of options may increase decision-rationality [10].

In 13.9% the problem was registered as both somatic and psychosocial where emphasis was given both to the symptom and the person. In these situations the GPs were more often uncertain and gradual and deliberate decision-making was most often registered. The diagnoses and wording of the rules of thumb registered illustrate these situations as more complex, demanding thorough anamneses, examination and individualised communication. Complex consultations are usually longer than straightforward ones [18,19].

## Conclusion

In conclusion, most patients consulted with somatic problems. The decision-making (immediate or gradual) registered by the GPs seemed to have been adjusted on the symptom or on the patient as a person. Immediate decision-making, which builds on recognition, was most frequent in somatic problems with emphasis given to symptoms and psychosocial problems with emphasis given to person in contrast to problems registered as somatic and psychosocial where emphasis was given both to the symptom and the person, where gradual decision-making was more often used in. Our results indicate that the GPs seem to recognise immediately both problems and persons, hence the quintessence of the expert skill of the GP as developed through experience.

## Competing interests

Authors declare that they have no competing interests.

## Authors’ contributions

MA, AA CER and LB made the conception and the design of the study, made the analyses and interpretations and revised the paper; all have given a final approval of the version to be published. MA and AA collected the data and did the main part of the writing.
